# Simulating and Predicting the Mechanical Behavior of Electrospun Scaffolds for Cardiac Patches Fabrication

**DOI:** 10.3390/ma16227095

**Published:** 2023-11-09

**Authors:** Elli Gkouti, Aleksander Czekanski, Ahmed AlAttar

**Affiliations:** Department of Mechanical Engineering, Lassonde School of Engineering, York University, Toronto, ON M3J1P3, Canada; aalattar@yorku.ca

**Keywords:** electrospinning, helical electrospun samples, viscoelastic behavior, stress softening, hyperelasticity, finite element analysis, simulations

## Abstract

Fabricating helical scaffolds using electrospinning is a common approach for cardiac implantation, aiming to achieve properties similar to native tissue. However, this process requires multiple experimental attempts to select suitable electrospun properties and validate resulting mechanical responses. To overcome the time and cost constraints associated with this iterative procedure, Finite Element Analysis (FEA) can be applied using stable hyperelastic and viscoelastic models that describe the response of electrospun scaffolds under different conditions. In this study, we aim to create accurate simulations of the viscoelastic behavior of electrospun helical scaffolds. We fabricated helical fibers from Poly (3-caprolactone) (PCL) using the electrospinning process to achieve this. The electrospun samples were subjected to uniaxial deformation, and their response was modelled using existing hyperelastic and stress relaxation models. The simulations were built on experimental data for specific deformation speed and maximum strain conditions. The FEM results were evaluated by accounting for the stress-softening phenomenon, which significantly impacted the models. The electrospun scaffolds’ predictions were performed in other than the initial experimental conditions to verify our simulations’ accuracy and reliability.

## 1. Introduction

Cardiovascular disease poses a global challenge that affects populations across the world. The fundamental issue lies in the fact that cardiomyocytes (cardiac muscle cells) cannot proliferate, and the heart contains a limited number of stem cells, rendering it incapable of self-regeneration. This deficiency leads to chronic cardiac dysfunction or even death [1]. The complexity of this issue arises from inadequate blood supply to the heart muscle, resulting in scar tissue formation and ventricular dilation [2]. Presently, the sole remedy for end-stage heart failure is heart transplantation, a resource-intensive and limited option. As a result, it is imperative to explore alternative medical treatments to repopulate the scar tissue on the heart with functional contracting cells to restore its proper function.

While the implantation of seeded scaffolds through stem cell therapy has limitations due to inefficient cell accumulation, tissue engineering offers an additional solution by using electrospinning to create extracellular matrix (ECM) scaffolds, facilitating the organization of cells into functional cardiac tissue for restoring heart function in cases of infarction [3,4]. Each application demands the fulfillment of specific criteria to achieve an optimally functioning seeded scaffold. In the context of cardiac applications, the mechanical properties and matrix architecture need to align with the inherent characteristics of native tissue, particularly concerning scaffold fibers and the electrospinning process [5,6].

An effective scaffold should mirror native tissue properties, including its often-overlooked mechanical attributes. Native cardiac tissue can expand up to 20% during contractions at around 200 beats per minute, a strain level not typically matched by straight-fiber scaffolds [7]. On the contrary, by developing helical or spring-like fibers, it is feasible to achieve similar stretching behavior with the cardiac tissue during muscle expansion and contraction [8,9], enabling the emulation of the heart’s cellular microenvironment, which, in turn, promotes cell contraction. Furthermore, their enhanced elasticity and extensibility significantly improve cardiac tissue function with stronger contractions, higher beating rates, and lower excitation thresholds than straight-fiber scaffolds. A recent study [9] showcased the successful creation of a 3D scaffold with spring-like fibers, closely mimicking the heart’s perimysial fibers, which play a crucial role in enabling the stretching and contraction of the myocardium, thus contributing to the development of functional cardiac tissue capable of generating robust contraction forces.

Moreover, synthetic-fiber scaffolds have higher stiffness (50 MPa–4000 MPa) than the native tissue (200 kPa–500 kPa) [9]. Native tissue experiences tension during blood intake (systole) and compression during pumping (diastole). Its intricate loading pattern is assessed with in vivo ultrasonics, revealing strain rates [10]. Rapid and frequent loading complicates scaffold property testing. Finally, note that the native tissue displays elastic behavior, as seen in unloading when the strain rate approaches zero [11].

Analytical models simulate complex material properties, which are challenging to examine physically. Various methods predict mechanical behaviors. Obtaining mechanical traits of spring-like electrospun scaffold aids model development. A model for a PCL-based straight-fibered scaffold was derived earlier, considering Fung’s Quasi-Linear Viscoelastic (QLV) and Arruda and Boyce’s eight-chain models [5]. The QLV applies to soft tissues, while synthetics show nonlinear, rate-dependent behavior [12]. The eight-chain model for elastomers was adapted for human skin [13]. The PCL scaffold exhibits complex viscoelastic behavior and stress softening. Regarding the latter phenomenon, it is recorded that stress decreases after applying repeating loading cycles due to molecular interactions [14,15,16,17]. Owning its characteristics to hyperplasticity and damage mechanics, it is required to precondition the samples before being subjected to any experimental test to reveal actual mechanical behavior [16]. In addition, stress softening can also be modeled using hyperelastic models, such as the Ogden model, by adding an extra term representing a damage function [18].

Obtaining the constitutive matrix for the material is a pivotal stage in ensuring the success of creating a scaffold for cardiac implantation. The initial step towards achieving this significant objective in tissue engineering involves comparing test data with established theoretical models. While utilizing uniaxial test data alone can yield accurate curve fittings, including various test configurations such as equibiaxial or shear tests is highly recommended to enhance the accuracy of the models. Moreover, the assumptions drawn before data evaluation play a critical role in refining the precision of the models when contrasted with material responses [16,19,20].

Achieving properties similar to the native cardiac tissue requires understanding the scaffold’s complex behavior. Multiple experimental iterations are necessary to accomplish this, which is time- and money-consuming. To overcome this insufficient repeating process, it is mandatory to create simulations that will provide us with accurate PCL scaffold predictions without repeating experimental tests whenever we need to examine different conditions. Besides other applications, FEM simulates human skin tissue using isotropic nonlinear elastic constitutive models [13]. However, the electrospun scaffolds’ complex behavior needs special attention to achieve adequate results.

In this study, we aim to develop a Finite Element Model (FEM) that can effectively predict various scenarios to which scaffolds might be exposed. To accomplish this, we produced fiber scaffold samples using the electrospinning technique and subsequently exposed these specimens to different strain levels. Given the viscoelastic behavior exhibited by the PCL samples, we characterized their reaction to varying strains by utilizing existing hyperelastic models for increasing strain and the Prony series for constant strain. Recognizing the importance of considering the stress-softening phenomenon for precise modeling, we preconditioned all samples before the primary experimental test. We constructed a FEM to anticipate the material’s response to lower strain levels during both the loading and relaxation phases by employing models designed for higher strain conditions as inputs. The outcomes of our simulations unequivocally demonstrate that achieving accurate predictions for the electrospun PCL samples is attainable if coupons devoid of stress-softening effects are considered.

## 2. Materials and Methods

### 2.1. Material and Sample Preparation with Electrospinning

Material selection strictly adhered to criteria that prioritize biocompatibility and bioabsorbability. Specifically, a biocompatible scaffold material should seamlessly integrate with cardiac tissue without inducing any adverse immune reactions or unfavorable consequences. Additionally, the chosen material’s bioabsorbable properties are beneficial because, as the scaffold initially supports tissue regeneration, it gradually breaks down in the body, ultimately leaving behind fully regenerated natural tissue. This eliminates the necessity for surgical removal or the persistence of foreign materials over the long term. Guided by these criteria, we adopted the optimal parameters derived from our prior sensitivity analysis [21,22]. Consequently, we opted for a Poly (3-caprolactone) (PCL) with an average molecular weight of 80,000. The solvents of choice, Dimethylformamide (DMF) and Dichloromethane (DCM), were both combined at a 15% concentration with the PCL. To ensure thorough mixing, the solution was stirred on a magnetic stirrer for 12 h in an air-sealed environment, preventing the volatile chemical DCM from evaporating. All materials were provided by Sigma-Aldrich, Oakville, ON, Canada.

**Figure 1:**
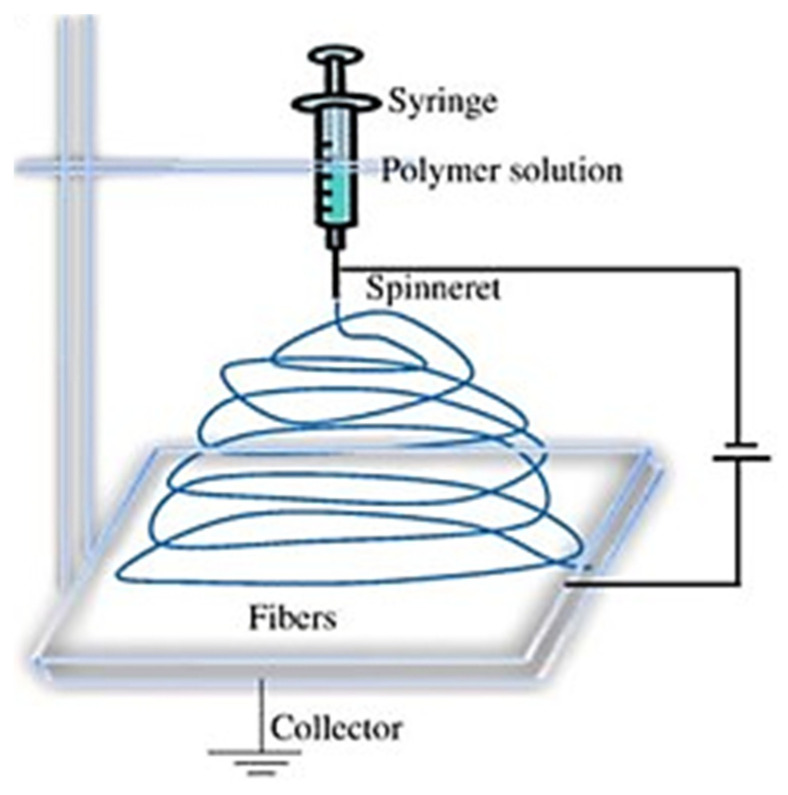
Scaffold fabrication scheme using an electrospinning process.

The resultant solution was carefully placed within a 5 mL syringe equipped with a 20 G needle and connected through a Luer lock mechanism to the NanoSpinner Electrospinning Device provided by Inovenso, Gambridge, MA, USA. Controlled dispensation was achieved using a syringe pump operating at a consistent 1 mL/h flow rate. The needle was connected to the positive terminal, while the negative terminal was affixed to an aluminum sheet, serving as the collector. Figure 1 illustrates the setup used for electrospinning in our experimentation. A constant voltage of 17.5 kV was maintained throughout all test runs. The distance between the tip and the collector (TCD) was fixed at 15 cm, and the collector was positioned at a 0-degree angle, as depicted in Figure 1. Upon the scaffolds’ completion of the drying process, preparations for creating tensile samples commenced. Surgical scalpels were used to carefully cut the samples, adhering to specific dimensions guided by a mold. The resulting tensile coupons measured 28 mm × 10.5 mm. Due to the inherent variability of the electrospinning process and its stochastic outcomes, the thickness of the samples exhibited variation among the individual tensile coupons. For the current study, we considered the average thickness of 0.08 mm. The selection of the electrospinning parameters was not random. On the contrary, it is an outcome of our previous detailed sensitivity study that led to the most accurate variables applicable for cardiac patch development, which has specific strict requirements (e.g., helical shape) [21].

Upon completing the manufacturing process, the samples were dried and cut into tensile rectangular coupons using a surgical scalpel in order to perform mechanical testing of their viscoelastic behavior. These fiber-based samples were 28 mm × 10.5 mm in size, controlled with a 3D printed mold, while their thickness was calculated as an average of 0.07 mm. Controlling the thickness in the electrospinning process is a drawback addressed in the literature [23,24] that we also faced in our previous study [21]. Variations in scaffold thickness were attributed to the randomness of fiber deposition and attempts to achieve uniform thickness by extending the electrospinning duration had negative consequences. An optical microscope (Zeiss Toronto, ON, Canada) and image analysis software (ImageJ) were used for thickness measurement.

### 2.2. Experimental Setup for Mechanical Testing

**Figure 2:**
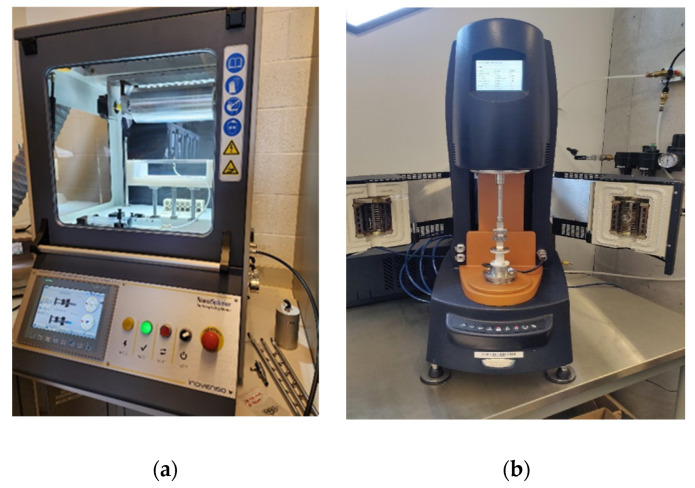
(**a**) Fabricating helical fiber samples using a NanoSpinner Electrospinning Device (Inovenso, Gambridge, MA, USA) and then subjecting them to (**b**) uniaxial tests using a TA Instruments Discovery Rheometer (TA Instruments Headquarters, New Castle, DE, USA) with the tensile fixture installed.

**Figure 3:**
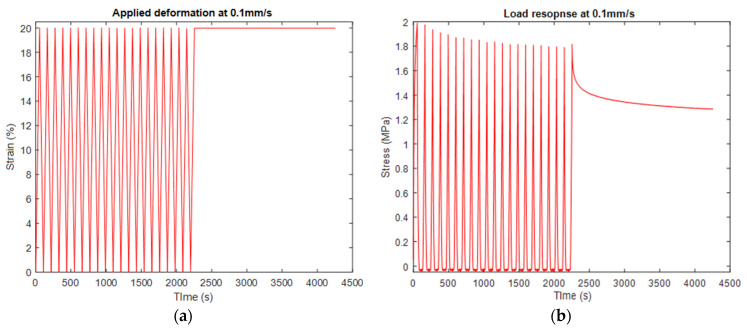
Uniaxial tensile test of electrospun samples subjected to 20 cycles of loading–unloading preconditioning before the actual experiment, which included a loading until reaching 20% strain and a relaxation stage at 0.1 mm/s speed. The results show the (**a**) strain vs. time and (**b**) stress vs. time. These results were used as an input for the FEM simulations.

To capture the hyperelastic behavior of the electrospun scaffolds, we subjected the fabricated samples to uniaxial tension until they reached a 20% strain, as depicted in Figure 2. As observed earlier, the electrospun scaffolds notably softened the required stress levels following their initial stretching. We conducted a repeated experiment involving coupon preconditioning to address this phenomenon, as illustrated in Figure 3. Specifically, we exposed the samples to a sequence of 20 loading–unloading cycles prior to subjecting them to the uniaxial tension test. To delve into the influence of deformation speed on the behavior of the samples, we replicated the same experimental setup using 0.1 mm/s speed. In pursuing a more precise model, we maintained a consistent strain for 2000 s, allowing us to capture the complete stress relaxation response. Consequently, introducing prior conditioning involving 20 cycles profoundly impacted the coupons’ hyperelastic and stress relaxation response. This effect was manifested in the modeling outcomes, resulting in potentially misleading conclusions concerning the accuracy and stability of the models employed in this study.

### 2.3. Modeling the Viscoelastic Behavior of Electrospun Scaffolds

#### 2.3.1. Mimicking the Mechanical Behavior of the Native Cardiac Tissue

Creating scaffolds for cardiac patches requires accurately mimicking the viscoelastic properties of the natural heart tissue. As mentioned above, the native tissue can expand by up to 20% of its resting size during the systolic phase when the heart is pumping blood rapidly at 200 beats per minute [25,26]. However, a significant concern arises when the scaffolds experience stress relaxation at high loads, mainly due to unpredictable plasticity phenomena. This issue becomes even more critical when we do not comprehensively understand the actual viscoelastic behavior because it is often masked by stress-softening and folding (plasticity) events, leading to a significant reduction in the required stress and an increase in deformation.

To tackle this challenge, various studies have suggested using cyclic preconditioning on the samples before integrating them with native tissue. This method effectively eliminates stress softening and folding, as discussed in references [5,27]. Consequently, applying preconditioning before use makes the viscoelastic characteristics of electrospun scaffolds apparent, revealing any potential limitations they may pose in developing cardiac patches.

#### 2.3.2. Transient and Stress-Softening Effects

In our previous study [21], we observed that this softening is mainly caused due to the viscoelastic behavior of the material and partially due to their plastic behavior since the PCL electrospun samples were recorded to recover almost 90% after 24 h resting from the experimental procedure completion (Table 1). Hence, it is mandatory to precondition all the samples before their exposure to any experimental procedure. In other soft materials with similar behavior, such as elastomers which exhibit this phenomenon (Mullins effect), there is an alternative to preconditioning samples. An extra term–damage function–in (existing or not) hyperelastic models (Ogden model) can account for the softening after prior cycles. However, some alternative loading–unloading cycle tests must be conducted to calculate the corresponding damage term parameters of the examined material’s sample. In the current study, we preconditioned every sample before their primary experimental test. Additionally, the existing hyperelastic models need to be further investigated for their accuracy in modeling the PCL electrospun samples’ behavior. As mentioned above, more complex models (e.g., Fung’s model) accounting for anisotropic responses might enhance the modeling accuracy.

Following the mechanical characterization of the scaffold, it became evident that additional tensile testing was necessary to gather data for modeling the behavior of preconditioned and non-preconditioned samples. The electrospun samples underwent the same loading protocol to achieve this, involving a 20% strain followed by 2000 s of sustained strain at 0.1 m/s speed. The chosen deformation level is intended to replicate the natural tissue’s ability to stretch up to 20% strain. The obtained results were subjected to meticulous analysis to derive hyperelastic and viscoelastic models. We performed the curve-fitting process in specialized commercial software (ABAQUS CAE). Numerous numerical models were explored to identify the most stable models for the hyperelastic portion. The acquired test results were utilized as uniaxial data in this endeavor, serving as a foundation for model calibration and validation.

### 2.4. Simulations and Predictions of the Viscoelastic Behavior of Electrospun Scaffolds

The experimental results shown in the next section clearly recorded the viscoelastic behavior and the softening phenomenon. However, the critical question was whether this behavior could be simulated and predicted to reduce the number of required experiments and electrospun sample fabrication, which is a highly time-consuming procedure also leading to non-consistent results due to the extremely sensitive nature of the electrospinning process. Hence, building the FEM for simulating the behavior of the scaffolds is mandatory for accelerating this research related to cardiac patch development. To predict the scaffolds’ response to uniaxial deformation identical to the experimental setup, considering the softening phenomenon’s impact on samples we also used ABAQUS CAE. We created a 3D solid component with the exact dimensions of the rectangular sample (measuring 28 mm × 10.5 mm with 0.07 mm thickness) employed in our experiments to simulate both the loading and relaxation stages. A mesh size of 0.5 was utilized to enhance precision, and the element type selected was C3D8R, which is suitable for most materials experiencing viscoelastic behavior exposed to large deformation. For defining the boundary conditions and steps in the FEM, we mimicked the experimental procedure; namely, one edge of our part was held constant while the other was extended with 0.1 mm/s speed until it reached 10% strain. Then, it was held constant at this level for 300 s. The steps used were static and visco for simulating the loading and stress relaxation stage.

Additionally, the boundary conditions closely mimicked those employed in the experiments, wherein one edge remained fixed while the other was permitted to extend freely. Furthermore, we used a “static” step to effectively simulate loading, whereas a “visco” step was incorporated for relaxation. The temporal duration of the “static” steps matched the constant strain rate achieved for 0.1 mm/s speed, while for the “visco” step, a period of 300 s. Although the relaxation performed during experiments was selected to be 2000 s, we reduced this time to 300 s for this study for computational efficiency. We assume that there is no impact on the accuracy of the results.

## 3. Results and Discussion

### 3.1. Experimental Tests

The stress softening is significant after the initial loading but continues even after the 17th cycle. After this cycle, the stress reached an approximately stable level. The behavior recorded in the 21st loading cycle was used to model the hyperelastic behavior of the samples considered preconditioned with the prior 20 loading–unloading cycles. From now on, the deformation scheme shown in Figure 3 will be regarded as uniaxial tension with “preconditioned” samples.

**Figure 4:**
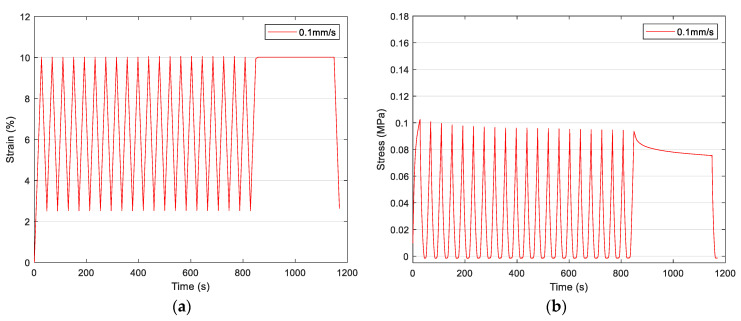
Uniaxial tensile test of electrospun samples subjected to 20 cycles of loading–unloading preconditioning prior to the actual experiment, which included a loading until reaching 10% strain and a relaxation stage at 0.1 mm/s speed. The results show the (**a**) strain vs. time and (**b**) stress vs. time. These results were used for evaluating the prediction of our FEM.

The results shown in Figure 3 were used as input for our FEM. Also, we used the curve-fitting procedure to model the sample’s viscoelastic behavior as recorded in the specific experiments. The results are shown in the next section. Figure 4 shows the corresponding experimental results for the case of 10% strain at 0.1 mm/s speed. These results were used for evaluating the predictions of the created FEM shown in the following sections.

It is known that materials exhibiting viscoelastic behavior, like our study’s electrospun scaffolds, exhibit different responses when increasing or decreasing the level of deformation. To prove this assumption, we also performed the same uniaxial experiment with preconditioned samples as shown in Figure 3 but with a reduced strain level of 10%. Stress softening also occurred, although its impact was recorded to be weakened. Another reason for reperforming this uniaxial test for 10% strain was to verify our developed FEM simulations and predictions.

### 3.2. Modeling Scaffolds’ Viscoelastic Behavior Results

The loading stage corresponds to an increased tension stress for the sample to achieve the desired strain level. This path is more complex to be explained with elasticity terms, so we use the nonlinear models of the hyperelasticity, including the strain energy density functions, to calculate the nominal stress. The strain energy density function can vary depending on the application, namely the material that needs to be described and its mechanical properties. In our study, we used hyperplastic models that accurately describe the scaffolds’ response to nonlinear deformation. The accuracy of these models depends on multiple factors such as the number of available experimental data, complex behavior like stress softening, etc. In our case, the stable hyperelastic models for preconditioned and non-preconditioned samples are shown in Table 2.

The strain energy density function of the models listed in Table 2, namely the Polynomial (*Pol*), Reduced Polynomial (*Red. Pol.*), Ogden of various terms, and Arruda–Boyce (*AB*) hyperelastic models are expressed with the following equations [16,18,27]:

(1) $$W_{Pol}=\sum_{i+j=1}^{N}C_{ij}{\left(I_{1}-3\right)}^{i}{\left(I_{2}-3\right)}^{j}+\sum_{i=1}^{N}\frac{1}{D_{i}}{\left(J-1\right)}^{2i},$$

(2) $$W_{Red.\;\;Pol}=\sum_{i=1}^{N}C_{i0}{\left(I_{1}-3\right)}^{i}+\sum_{i=1}^{N}\frac{1}{D_{i}}{\left(J-1\right)}^{2i},$$

(3) $$W_{Ogden}=\sum_{i=1}^{N}\frac{2\mu_{i}}{a_{i}^{2}}(\lambda_{1}^{a_{i}}+\lambda_{2}^{a_{i}}+\lambda_{3}^{a_{i}})+\sum_{i=1}^{N}\frac{1}{D_{i}}{\left(J-1\right)}^{2i},$$

(4) $$W_{AB}=\mu \left[\frac{1}{2}\left(I_{1}-3\right)+\frac{1}{20\lambda_{m}^{2}}\left(I_{1}^{2}-9\right)+\frac{11}{1050\lambda_{m}^{2}}\left(I_{1}^{3}-27\right)+\frac{19}{7000\lambda_{m}^{2}}\left(I_{1}^{4}-81\right)+\frac{519}{673750\lambda_{m}^{2}}\left(I_{1}^{5}-243\right)\right]+\frac{1}{D}\left(\frac{J^{2}-1}{2}-\ln J\right),$$

where *N* is the number of terms required to model the hyperelastic behavior accurately. $I_{1},\;I_{2}$ are the two invariants of the left Cauchy–Green deformation tensor, $\lambda_{i}$ (i=1, 2, 3) are the stress ratio for each direction, J is the Jacobin for volume changes, and $D_{i}$ are the material incompressible parameters, which in our study is zero as the material is considered incompressible. For Polynomial (1) and Reduced Polynomial (2) models, $C_{ij}$ (i,j=1, 2, 3) are the material coefficients required to be determined. For the Ogden model, $\lambda_{i},\;a_{i}$ (i=1, 2, 3) are the material coefficients, while $\mu ,\;\lambda_{m}$ (m=1, 2, 3) correspond to Arruda–Boyce model.

It must be noted that the Reduced Polynomial hyperelastic model is essentially a specific type of Polynomial model where the complexity of the polynomial function is reduced by making certain simplifications or assumptions. These simplifications can involve limiting the number of terms in the polynomial expansion or assuming that some material parameters are zero. Furthermore, the Reduced Polynomial is often employed when the material behavior can be reasonably approximated with a more straightforward mathematical form. This simplification can make the model computationally more efficient. Hence, both Polynomial and Reduced Polynomial models are used to describe the hyperelastic behavior of soft materials like our case of electrospun scaffolds, with the critical difference being the level of complexity and detail in the mathematical representation. The choice between the two depends on the specific material behavior modeled using a curve-fitting process with experimental data.

The assessment of hyperelastic model stability is based on the Drucker stability criterion, which suggests that a material model is considered stable when the strain energy related to the incremental stress exceeds zero.

Results without accounting for the stress softening provide insufficient models, which cannot account for deformation beyond the provided deformation range, leading to the model’s instabilities. It must be noted that the reduced number of stable models is also a result of having only one input test data, the uniaxial tension in our case. If more tension tests were performed (e.g., shear, equibiaxial), the number of stable models would have been increased even for the non-preconditioned samples. However, it is known that the complex phenomenon of stress-softening significantly impacts hyperelastic behavior that cannot be captured and predicted accurately.

**Figure 5:**
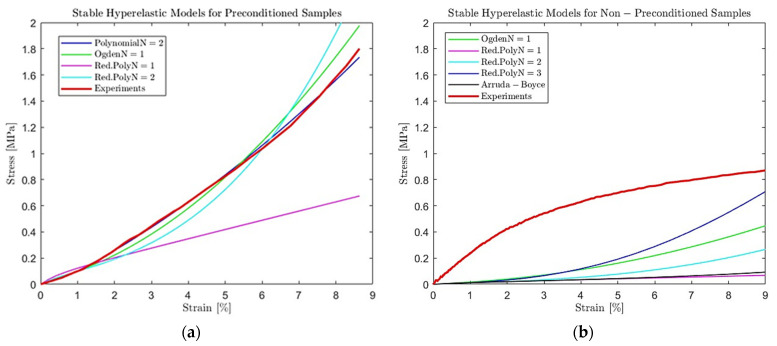
Modeling the viscoelastic behavior of (**a**) preconditioned and (**b**) non-preconditioned electrospun samples subjected to 10% strain with 0.1 mm/s using the curve-fitting process of stable hyperelastic models compared to experimental uniaxial tensile test data.

All of the stable models, shown in Figure 5, with an adequate curve fitting with the experimental data, can be used to model the hyperelastic behavior of electrospun samples. The models could be used for linear curves if the predictions concern small deformations, namely less than 10–15% strain. The hyperelastic behavior is nonlinear for large deformation, and more terms must be added to the models. Thus, in our study, we used the Polynomial model with two terms (*N* = 2) to model the loading stage of our electrospun samples. The material coefficients for the Polynomial model with *N* = 2 were calculated in Abaqus following the curve-fitting process and are shown in Table 3.

Additionally, for modeling stress relaxation, we selected to use the Prony series expressed by the following relationship:

(5) $$\sigma_{R}(t)=\left[1-\sum_{t=1}^{N}g_{i}(1-e^{-t/\tau_{i}})\right],$$

where $\sigma_{R}$ is the normalized stress regarding the initial stress that the stress relaxation begun and $g_{N},\;\tau_{N}$ are the material coefficients. N is the number of terms that are required to be used in order for the model to provide adequate results.

**Figure 6:**
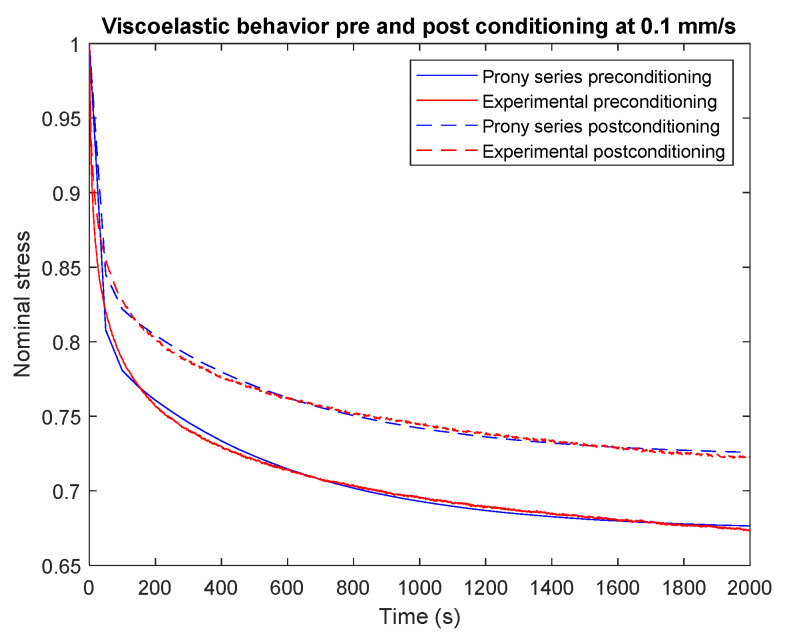
Modeling the stress relaxation behavior of preconditioned vs. non-preconditioned electrospun with 0.1 mm/s speed using the curve-fitting process compared to experimental stress relaxation test data.

The number of terms n of the Prony series was determined in Abaqus. The factors affecting it are the accuracy specified by the average root-mean-square error (0.001 in our case), the stability of predicting other loading/displacement conditions and the computationally efficient number of terms. Hence, we used two terms (N=2) of the Prony series, where their coefficients (Table 3) were calculated in Abaqus using the curve-fitting approach with test data and the results for both cases are shown in Figure 6.

### 3.3. Simulating Scaffolds’ Viscoelastic Behavior

Since the PCL electrospun samples exhibit both viscoelastic and plastic behavior, the scaffolds exhibit a softening phenomenon after the initial stretching of the specimen. As a result, when the material is exposed to multiple tension, the stress level is degraded by almost 95% compared to the initial loading–unloading cycling (Table 1). This behavior impacts the loading/unloading stage, where the stress is gradually increased/decreased with increasing strain (nonlinear behavior when exposed to high deformation) and the stage where deformation is held constant after loading/unloading. The material’s response to this cause is stress relaxation/recovery.

The importance of preconditioning samples and accounting for the stress-softening phenomenon is evident in the results in Figure 3 and Figure 4. In this case, we used as an input the experimental results recorded for preconditioned samples subjected to uniaxial tension with 0.1 mm/s speed until they reached 20% strain. The preconditioning was held for 20 loading–unloading cycles until 20% strain was reached. The selected hyperelastic model was Polynomial *N* = 2 since the curve-fitting procedure provided the most accurate results, as shown in Figure 5. As an input, we used the parameters of this model shown in Table 3. Using FEA, we built simulations of the scaffolds’ behavior when exposed to uniaxial tension until 10% strain was reached with a deformation speed of 0.1 mm/s.

**Figure 7:**
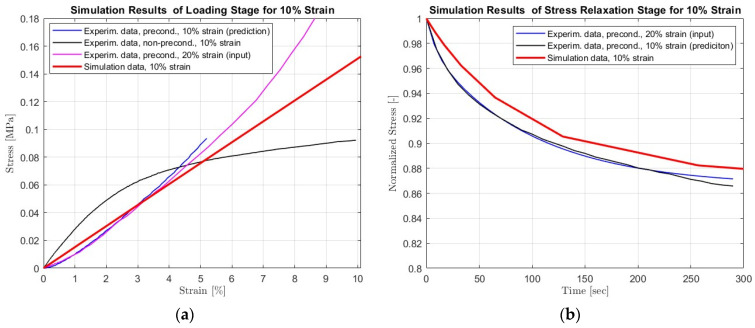
Simulation vs. experimental test results of the scaffold subjected to (**a**) uniaxial tensile and (**b**) stress relaxation with 0.1 mm/s speed. The data used as an input corresponds to preconditioned samples (no stress softening) subjected to 20% maximum strain.

The results show an adequate simulation of the loading stage with the preconditioned samples (less than 10% deviation). In contrast, our simulation failed to predict the corresponding response of the virgin scaffolds where no mechanical testing operation was performed on them (no preconditioning). It must be noted that the simulation results shown in Figure 7 can be further improved. One way to proceed is to perform more tests capturing the shear and equabiaxial behavior of the samples. Hence, the input test data will enhance the hyperelastic models’ accuracy since they will be built on not only one but three tensile tests. Following this path, the models’ stability and accuracy will be enhanced overall.

As explored from the previous results, the loading stage is mainly impacted by the softening phenomenon compared to the stage where the deformation is held constant, and stress relaxation occurs in the scaffold. The simulation results of this stage are presented in Figure 7, which show an enhanced accuracy. To build a FEM simulating this stage, we performed experimental tests shown in Figure 3. After reaching 20% strain with tension at 0.1 mm/s speed, the stress relaxation test was performed. The constant deformation was stable for 2000 s, and the samples’ response was recorded. Then, the experimental data was modeled using the curve-fitting method with the Prony series. The calculated coefficients are shown in Table 3, which were used as input for the developed FEM simulating the stress relaxation stage. The developed FEM was used to predict the scaffolds’ response to stress relaxation after applying uniaxial tension with 0.1 mm/s and reaching 10% strain. For sufficient computational time, we simulated the relaxation stage for 300 s. We assume that no impact in the building model occurred due to this reduction. The simulation results for this stage are shown in Figure 7. The results show an enhanced accuracy (less than 10% deviation) in predicting stress relaxation at 10% strain. Compared to the simulations in the loading stage, this phenomenon slightly affects stress relaxation. However, stress relaxation is a sequel stage from the loading, which is highly impacted by this phenomenon. Thus, non-consideration of this effect will impact the hyperelastic models, leading to misleading simulations and predictions of the overall viscoelastic behavior of the electrospun scaffolds.

## 4. Conclusions

The fabrication of helical scaffolds through the electrospinning technique remains a prevalent strategy for cardiac implantation, intending to achieve properties that closely resemble native tissue. However, this process involves multiple iterative experimental trials to identify appropriate electrospun characteristics and validate ensuing mechanical responses. To surmount the associated time and cost constraints, applying Finite Element Analysis (FEA) using stable hyperelastic and viscoelastic models has emerged as a valuable solution. These models comprehensively describe the behavior of electrospun scaffolds across diverse conditions. In the current study, our primary focus was creating precise simulations to capture the viscoelastic behavior exhibited by electrospun helical scaffolds. Our approach encompassed fabricating helical fibers from the PCL through the electrospinning process. These electrospun samples underwent uniaxial deformation, and their reactions were modeled using pre-existing hyperelastic and stress relaxation models. These simulations were constructed based on experimental data acquired under specific conditions, encompassing deformation speed and maximum strain. However, the influence of the stress-softening phenomenon constitutes a significant factor impacting the models and, consequently, the simulations. Hence, the present study was carefully assessed in evaluating the curve-fitting and FEM results. We applied electrospun scaffold behavior predictions other than the initial experimental conditions to verify our simulations’ accuracy and reliability. Subsequently, we developed a comprehensive FEM capable of simulating the performance of electrospun specimens, facilitating predictions of relevant outcomes under varying conditions. The successful simulation of these responses underscores the ability to accurately model the loading and stress relaxation stage using viscoelastic theory, effectively eliminating the stress-softening phenomenon. The employment of commercial software utilizing time-domain constitutive equations of viscoelasticity played a pivotal role in achieving these precise simulations. Ultimately, the successful integration of FEA provides invaluable insights into the mechanical behavior of electrospun scaffolds, fostering a comprehensive understanding and predictive capability concerning their performance.

## Figures and Tables

**Table 1:** Length change after subjecting the sample to uniaxial tension to preconditioning (20 cycles) and 24 h resting.

Condition	Length (mm)
Initial length	28 mm
Deformed (after testing completion)	31.2 mm (11.6% from the initial length)
Recovered (24 h resting)	29.7 mm (6% from the initial length)

**Table 2:** Stable hyperelastic models for preconditioned compared to non-preconditioned electrospun samples.

Hyperelastic Models	Preconditioned Samples	Non-Preconditioned Samples
Polynomial *N* = 2	✓	-
Ogden *N* = 1	✓	✓
Red. Polynomial *N* = 1	✓	✓
Red. Polynomial *N* = 2	✓	✓
Red. Polynomial *N* = 3	-	✓
Arruda–Boyce	-	✓

**Table 3:** Polynomial (*N* = 2) and Prony series (*N* = 2) coefficients for modeling preconditioned samples’ loading stage (hyperelastic) and stress relaxation stage, respectively, at 0.1 mm/s speed.

Polynomial Model Parameters		Prony Series Parameters	
C_10_	−0.02873762	G_1_	0.0226086
C_20_	0.00002634	G_2_	0.11157
C_01_	0.05022618	τ_1_	8.8895
C_11_	0.00101692	τ_2_	97.497
C_02_	0.02451267		

## Data Availability

Data are contained within the article.
